# Scales of Spatial Heterogeneity of Plastic Marine Debris in the Northeast Pacific Ocean

**DOI:** 10.1371/journal.pone.0080020

**Published:** 2013-11-20

**Authors:** Miriam C. Goldstein, Andrew J. Titmus, Michael Ford

**Affiliations:** 1 Scripps Institution of Oceanography, University of California San Diego, La Jolla, California, United States of America; 2 Marine Sciences Program, Hawai'i Pacific University, Kaneohe, Hawai'i, United States of America; 3 National Marine Fisheries Service, National Oceanographic and Atmospheric Administration, Silver Spring, Maryland, United States of America; University of Waikato (National Institute of Water and Atmospheric Research), New Zealand

## Abstract

Plastic debris has been documented in many marine ecosystems, including remote coastlines, the water column, the deep sea, and subtropical gyres. The North Pacific Subtropical Gyre (NPSG), colloquially called the “Great Pacific Garbage Patch,” has been an area of particular scientific and public concern. However, quantitative assessments of the extent and variability of plastic in the NPSG have been limited. Here, we quantify the distribution, abundance, and size of plastic in a subset of the eastern Pacific (approximately 20–40°N, 120–155°W) over multiple spatial scales. Samples were collected in Summer 2009 using surface and subsurface plankton net tows and quantitative visual observations, and Fall 2010 using surface net tows only. We documented widespread, though spatially variable, plastic pollution in this portion of the NPSG and adjacent waters. The overall median microplastic numerical concentration in Summer 2009 was 0.448 particles m^−2^ and in Fall 2010 was 0.021 particles m^−2^, but plastic concentrations were highly variable over the submesoscale (10 s of km). Size-frequency spectra were skewed towards small particles, with the most abundant particles having a cross-sectional area of approximately 0.01 cm^2^. Most microplastic was found on the sea surface, with the highest densities detected in low-wind conditions. The numerical majority of objects were small particles collected with nets, but the majority of debris surface area was found in large objects assessed visually. Our ability to detect high-plastic areas varied with methodology, as stations with substantial microplastic did not necessarily also contain large visually observable objects. A power analysis of our data suggests that high variability of surface microplastic will make future changes in abundance difficult to detect without substantial sampling effort. Our findings suggest that assessment and monitoring of oceanic plastic debris must account for high spatial variability, particularly in regards to the evaluation of initiatives designed to reduce marine debris.

## Introduction

Plastic debris has been documented in a wide variety of marine ecosystems, including the coastlines of remote islands, the coastal water column, the deep sea, and subtropical gyres [1]–[3]. Environmental impacts of large pieces of debris ranging from centimeters to tens of meters in diameter, termed “macroplastic,” include habitat alteration and damage, entanglement, and ingestion by megafauna such as cetaceans, seabirds, and sea turtles [4], [5]. Colonization of floating debris may also transport rafting species, leading to bioinvasions [6]. Environmental impacts of small plastic particles less than 5 mm in diameter, termed “microplastic,” include ingestion, accumulation of toxins, and alteration of the pelagic habitat through the addition of hard substrate [7].

Plastic pollution has rapidly increased over the past several decades [3]. Floating plastic was first documented in the North Pacific and North Atlantic subtropical gyres in the early 1970s, with observations of both microplastic [8]–[10] and large objects such as bottles [11]. Plastic debris abundance increased between the late 1960s through the 1990s as documented by at-sea surveys [12], [13], seabird ingestion studies in the Arctic and Atlantic [14], [15], a Continuous Plankton Recorder study in the northeast Atlantic [16], and coastal deposition on remote islands [17]. Since the 1990s, there is some question as to whether plastic density has continued to increase, since high spatial and temporal heterogeneity make shorter-term trends difficult to discern [18].

The spatial distribution of plastic marine debris is influenced by multiple interacting factors. Locally, wind patterns affect the distribution of debris by differentially moving or mixing particles of different densities [19], [20], and higher densities of debris in coastal waters can be associated with human population centers [21], [22]. Over regional scales, convergences such as the North Pacific Subtropical Convergence Zone and the Kuroshio Extension Recirculation Gyre collect debris [23], [24]. Over ocean basins, spatial patterns of debris are influenced by large-scale atmospheric and oceanic circulation patterns interacting, leading to particularly high accumulations of floating debris in the subtropical gyres [25], [26].

The spatial distribution of plastic marine debris is influenced by multiple interacting factors. Locally, wind patterns affect the distribution of debris by differentially moving or mixing particles of different densities [19], [20], and higher densities of debris in coastal waters can be associated with human population centers [21], [22]. Over regional scales, convergences such as the North Pacific Subtropical Convergence Zone and the Kuroshio Extension Recirculation Gyre collect debris [23], [24]. Over ocean basins, spatial patterns of debris are influenced by large-scale atmospheric and oceanic circulation patterns interacting, leading to particularly high accumulations of floating debris in the subtropical gyres [25], [26].

Assessing the spatial distribution of debris is important to understanding its environmental significance. Therefore, our objective in this study was to examine the abundance and variability of debris during two cruises in the eastern North Pacific. To this end, we asked the following questions: 1) What is the spatial abundance and distribution of pelagic microplastic in comparison to biophysical variables? 2) What is the size-frequency spectrum of oceanic plastic, and how does methodology affect estimates of its abundance?

## Materials and Methods

### Study area

**Figure 1:**
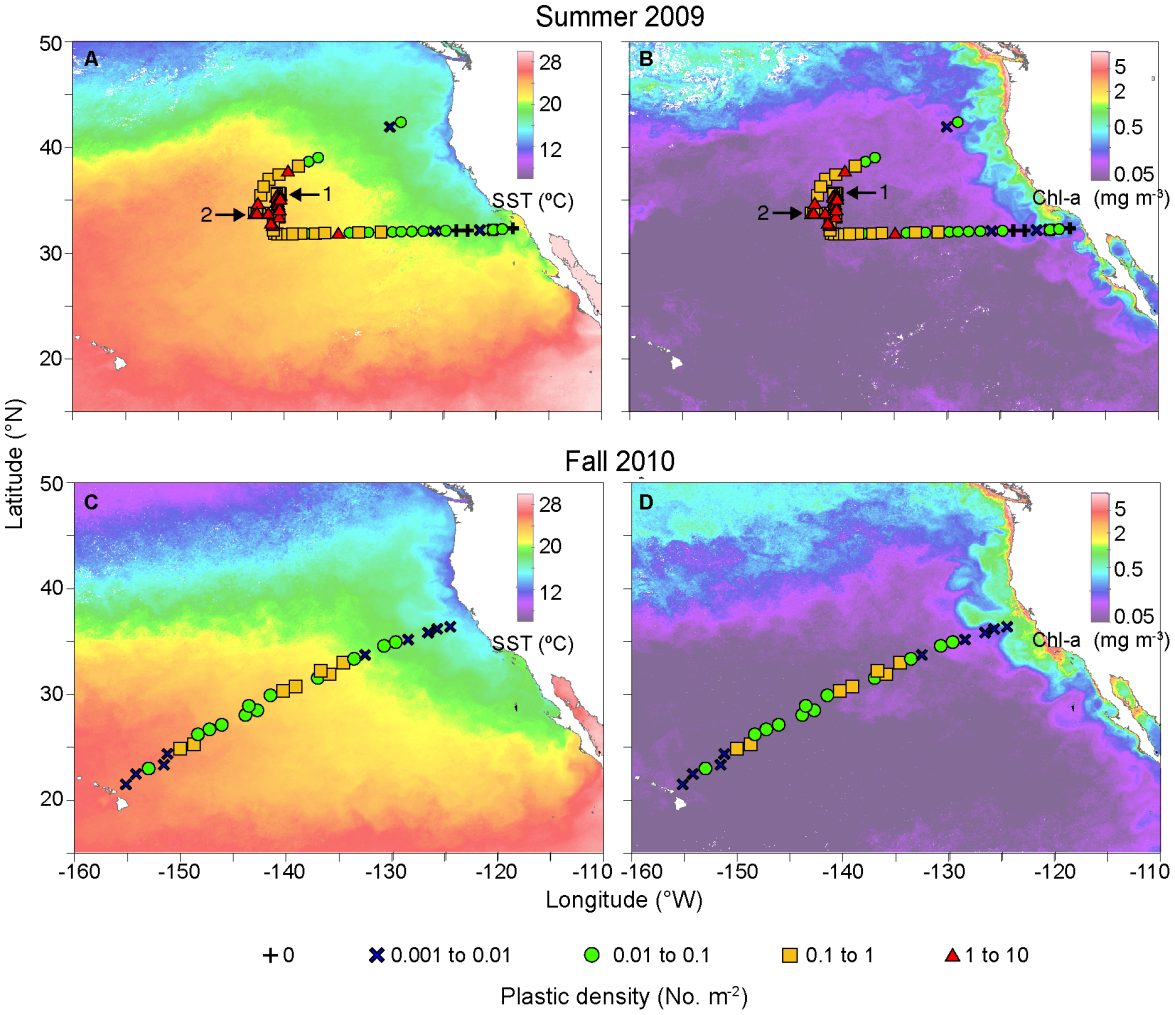
Microplastic numerical abundance superimposed on sea surface temperature and chlorophyll-*a*. A) Microplastic numerical abundance and sea surface temperature in Summer 2009 (n = 119); B) Microplastic numerical abundance and chorophyll-*a* in Summer 2009 (n = 119); C) Microplastic numerical abundance and sea surface temperature in Fall 2010 (n = 28); D) Microplastic numerical abundance and chlorophyll-*a* in Fall 2010 (n = 28). Symbols indicate the location of each manta tow, and microplastic numerical abundance is given in No. m^−2^. The locations of submesoscale sampling schemata are indicated by (1) grid sample; and (2) line sample. Temperature maps are monthly composites of MODIS-Aqua and MODIS-Terra data, and chlorophyll-*a* maps are monthly composites of SeaWiFS Level-2 data. Maps were created from composites of the calendar month in which each cruise took place (August 2009 and October 2010). White pixels denote no data due to cloud cover.

This study focused on a subset of the eastern North Pacific between the west coast of the United States and Hawaii, between approximately 20–40°N, 120–155°W. This area was selected for two reasons: a) proximity to the North Pacific Atmospheric High, under which debris is thought to collect; and b) ship time logistics. Cruise tracks and sampling locations are given in Figure 1.

### Net samples

From 2–20 August 2009, samples (n = 119) were collected on the Scripps Environmental Accumulation of Plastic Expedition (SEAPLEX) cruise on the R/V *New Horizon*. SEAPLEX samples presented here include: manta tows taken at predetermined times, manta and bongo tows taken on station, and visual observations of macrodebris taken between stations. SEAPLEX sampling also includes the manta tows and visual observations described in the *Submesoscale sampling schemata* section below. For manta tows taken at predetermined times, a single tow was taken either every 6 hours (ship local time 0300, 0900, 1500, 2100) or every 3 hours (ship local time 0000, 0300, 0600, 0900, 1200, 1500, 1800, 2100) depending on the ship time available. Because the ship traveled at 18.5 km hr^−1^, these tows were spaced approximately 74–111 km apart.

Three stations were selected to target high-plastic areas in the NPSG, and were compared to one reference station in the California Current off San Diego. The high-plastic stations were selected using visual observations and tow results, and the reference station was pre-selected. While on station, surface (manta) tow (n = 8 per station) and subsurface (bongo) tows (n = 6 per station) were performed as close to station coordinates as possible. Both types of tow were evenly split between day and night. Throughout this cruise, conditions in the NPSG were calm and glassy (Beaufort Sea State 1–2), and conditions in the California Current were also mild (Beaufort Sea State 3–4).

From 19–29 October 2010, samples (n = 28) were collected on the EX1006 leg of the Always Exploring Expedition on the NOAA ship *Okeanos Explorer*. Samples from this cruise include only manta tow samples taken at predetermined times (ship time 0600, 1200, and 1800) during daylight hours. Conditions were mild to moderate, ranging from Beaufort Sea State 2 off Hawaii, to 5 off California.

Manta tows on both cruises were collected using a standard manta net (0.86×0.2 m) with 333 µm mesh towed for 15 minutes at 0.7–1 m s^−1^ [27]. Water volume flowing through the net was measured with a calibrated General Oceanics analog flowmeter. The manta net samples the two-dimensional air-sea interface so concentrations are preferentially given in square meters, but when conversion to cubic meters is necessary, the depth sampled is assumed to be the 0.2 m dimension of the net opening [28]. The SEAPLEX samples were fixed in 1.8% formaldehyde buffered with sodium borate, and the EX1006 samples were fixed in 95% ethanol. No specific permissions were required for samples, since they were taken in federal or international waters and did not involve protected species.

Subsurface samples were collected on SEAPLEX using a CalCOFI bongo net (pair of circular frames 71 cm in diameter, 202 µm mesh). The bongo net was deployed in an oblique tow with a maximum depth of 210 meters for 15 minutes and retrieved at a constant speed, which ensures equal time at each depth and represents an integrated measure of the water column. As with the manta tow, water through the net was measured with a calibrated flowmeter. Upon retrieval the nets were washed carefully and the contents of one net preserved in 1.8% formaldehyde buffered with sodium borate. Six repeated bongo tows were conducted per station for a total of n = 24.

Each net tow sample was sorted for microplastic at 6–12× magnification under a Wild M-5 dissecting microscope, and plastic particles removed for further analysis. A subset of plastic particles (n = 557) were analyzed using Fourier transform infrared spectroscopy and all were confirmed to be plastic. Visual inspection of the plastic particles in this study was therefore deemed to be sufficient to confirm plastic identity.

Plastic particles were soaked in deionized water to remove salts, dried at 60°C, and stored in a vacuum desiccator. Dry mass was measured on an analytical balance. Particles were then digitally imaged with a Zooscan digital scanner with a resolution of 10.6 µm [29], [30]. The total number of particles as well as two-dimensional surface area and maximum diameter were measured using NIH ImageJ-based tools in the Zooprocess software, and calibrated against manual measurements [29], [30].

Dry mass of zooplankton was obtained from preserved manta tow samples [31]. After fixation in 1.8% formaldehyde for 24 months, samples were split in a Folsom splitter, filtered onto 202 µm Nitex mesh disks and rinsed with isotonic ammonium formate. Filters were dried for 24 hours at 60°C and placed in a vacuum dessicator until weighing. Filters were weighed to the nearest 0.0001 gram on the same analytical balance as the plastic samples. A 20% correction factor was applied in order to compensate for the biomass lost by preservation [31], [32]. Ratios were calculated by dividing microplastic dry mass by zooplankton dry mass.

### Visual observations

Visual observer counts for macrodebris on SEAPLEX were conducted by a single observer (A.J.T.) on one side of the flying bridge at 10 m eye height above sea level, while the vessel was transiting between stations. Because of the great differences in ship speed between transit areas and station areas, visual counts were limited to the transit areas only. Visual counts do not directly coincide with towed net samples (on station) because the visual counts were originally intended to be compared with marine bird counts [46].

The observer surveyed on one side of the track-line, based on sighting conditions (e.g., glare and wind). Sampling followed standardized line distance methods as follows in order to produce more accurate abundance estimates [33]. All marine debris sighted to the horizon was counted and assigned to one of seven pre-determined distance bins based on perpendicular distance from the ship. The distance bins used were 0–10, 10–50, 50–100, 100–200, 200–300, 300–600 and >600 m. Distance to the observed object was determined using a hand held range finder which consisted of a rod marked with the distance bins so that when held with its end up to the horizon would allow for a visualization of the distance bins [34]. Observed debris was also assigned to one of three pre-determined size classes based on its larger dimension: small (2–10 cm), medium (10–30 cm), large (>30 cm). Only three size classes were chosen because of the large amount of material observed and the need to be able to quickly classify and record it in order to allow for greater detection probability.

The color of each piece and a description (material type, dimensions) were also recorded. The favorable environmental and ocean conditions during the cruise allowed for maximum detection of debris size and color. Sizes of debris objects were determined from comparisons with debris pieces of known size, such as tubing, bottle caps or buoys and the longest dimension recorded. To convert observed diameter into two-dimensional area, visually observed objects were assumed to be circular. While describing observed debris based on its longest dimension is not ideal due to the irregular shapes of most pieces of debris, this method was deemed appropriate based on the volume of debris being sighted and has been used in other visual observation studies [21]. Of the items observed, 95.5% were solid plastic. Line, and polystyrene made up the majority of the rest of the 4.5% and only a few pieces were identified as glass, wood, or other. These were excluded from the analysis.

The distance data were used to determine the effective strip width (ESW) [33] for each type of debris based on size and color using DISTANCE 6 software [35]. We considered three size classes (small, medium, large) and three color classes (white, high visibility, low visibility). Because of the favorable environmental conditions during this cruise we were able to use all marine debris sightings throughout the entire range of sea states encountered (Beaufort range 0–4) after determining that there was no significant decrease in detectability with increasing sea state [36].

We next calculated the detection function using the model which best described the sighting distance distribution and minimized Aikake's Information Criteria (AIC). Then, we calculated a correction factor which was used to standardize the apparent densities of each of the marine debris groups to the group with the widest ESW [37]. The total number of sightings for each debris group was multiplied by its correction factor to create a corrected number of sightings, and densities for each group were determined by dividing the corrected number of sightings by the effective area surveyed (survey distance×maximum ESW). Distance traveled was determined using the GPS locations of the ship at the start and end of each survey section. Additional details on visual counts can be found in Titmus & Hyrenbach 2011 [36].

Visual measurements were matched with net tow measurements taken within 25 km of each other (n = 23 pairs). Because both sets of samples were taken along the cruise track transect, these are point measurements without replication, with the exception of the submesoscale sampling schemata described below.

### Submesoscale sampling schemata

Two submesoscale sampling protocols (stations spaced at 10 and 18 km) were used on the SEAPLEX cruise. These sampling protocols, done in addition to the net samples above, were designed to examine plastic abundance and variability on a smaller scale (10 s of km) than on the mesoscale (100 s–1000 s of km) cruise-track-wide sampling described above. First a grid pattern was deployed on August 12, 2009 centered around 30°48.6′N, 139°45.9′W. Designed to examine microplastic spatial variation on a 40 km grid, it consisted of 16 manta tows taken 10 km apart in a 4 by 4 grid pattern. The second was a line pattern deployed on August 15, 2009 proceeding west from 34°3.4′N, 141°22.4′W. Designed to compare microplastic and macroplastic abundance and variance, the line consisted of 4 stations of 5 repeated manta tows, for a total of 20 manta tows. The four stations were 18 km apart. Visual transect sampling of plastic macrodebris was performed between tow stations. To compare visual observations with net tow observations, visual observations were combined in over-the-ground bins of 900 meters in length. Due to calm conditions, the average net tow also covered 900 meters of over-the-ground distance. Macrodebris observations for 9 km on either side of a tow station were associated with the net tows from that station.

### Oceanographic context

Sea surface temperature was mapped over the study area using remotely sensed data from MODIS-Aqua and MODIS-Terra. Maps were created from monthly composites of the calendar month in which each cruise took place (August 2009 and October 2010). Chlorophyll was mapped using monthly composites of SeaWiFS Level-2. Both sets of maps were created by Mati Kahru (Scripps Institution of Oceanography, UCSD) [38]. These satellite images were used post-cruise to assign sampling stations to a water mass (California Current, transition region, North Pacific Subtropical Gyre) for the purposes of comparing the ratio of plastic to zooplankton biomass. Since the water mass assignments are based solely off surface data, they should be viewed as highly approximate.

True wind data were collected on the R/V *New Horizon* during SEAPLEX using an RM Young 85000 ultrasonic anemometer mounted in the starboard side of the ship's superstructure 11 m above the waterline. Data were downloaded from the Scripps Institution of Oceanography MetAcq System where true wind was derived from ship heading, course over ground, speed, and relative wind speed [39]. True wind data were collected on the NOAA Ship Okeanos Explorer during EX1006 using an RM Young 05106 aerovane mounted atop the ship's superstructure 17.7 m above the waterline. Data were downloaded from the ship SCS data system where true wind was derived from ship heading, course over ground, speed, and relative wind speed [39].

To compare the particle concentration to true wind speed, the microplastic particle concentration anomaly (from particles collected in manta tows) was compared to the true wind speed anomaly for each cruise. The anomaly was the difference between individual measurements of the particle concentration and the overall mean particle concentration, or between the individual true wind speed measurements and the mean true wind speed for the entire time series. Wind speed data recorded during particle sampling (manta net tows) were extracted from the full record of true wind data from each cruise and used in these analyses.

Fisch (2010) documented potential differences in wind speed between the sensor types used in these two cruises, finding that the ultrasonic anemometer measurements can be 0.3 m s^−1^ faster than the aerovane for average speed and 1.0 m s^−1^ faster at maximum speeds [40]. We sampled a range of wind speeds within the average wind speeds experienced in the Fisch study. Therefore, while intercalibration between the two ships was not conducted, it is assumed that it may be possible that the ultrasonic anemometer data from the SEAPLEX cruise may be 0.3 m/s faster than the aerovane data from the EX1006.

### Statistical analysis

The semivariogram, often abbreviated variogram, describes how data covary with distance, and can reveal large-scale spatial patterns in highly variable data. [41]. Semivariogram interpretation is based on the principle that pairs of samples that are closer to each other are more similar than pairs of samples farther apart. The semivariogram function should therefore increase with distance [42], [43]. Above a certain distance, sample pairs may no longer be correlated, and the semivariogram function may reach a steady value, or “sill.”

We used semivariograms to compare the spatial distribution of microplastic to standard biophysical variables of temperature, salinity, and chlorophyll-*a* fluorescence for the Summer 2009 dataset. Semivariograms could not be calculated for Fall 2010 due to an insufficient sample size. The standard empirical semivariogram is calculated as
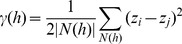
(1)where *N(h)* is the set of all pairwise Euclidean distances *i - j = h*, |*N(h)*| is the number of distinct pairs in *N(h)*, and *z_i_* and *z_j_* are data values at spatial locations *i* and *j*, respectively [41]. Because the standard semivariogram equation is sensitive to skewness in the data, we instead calculated the empirical semivariogram using the robust semivariogram estimator [44]. This method is based on the square root of the absolute value of the data value differences, *|z_i_−z_j_|^1/2^*, rather than the squares of the differences.
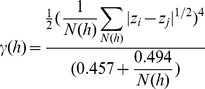
(2)Semivariograms were computed with the geoR package (version 1.7-1) [45] and fitted with either a linear or Gaussian model.

We conducted a power analysis on the Summer 2009 dataset to estimate the sample sizes that would be necessary to detect changes in microplastic abundance. This was calculated by multiplying the Summer 2009 dataset (n = 119) by a factor of increase (e.g., 20%, 30%, etc.), then using Monte Carlo simulations (1,000 simulations per test) to determine if the Mann-Whitney U test could detect the plastic increase with 95% confidence [46]. The power was determined by the percentage of the Monte Carlo simulations with a p-value of less than 0.05 – for example, if the null hypothesis was rejected in 80% of the simulations, the power would be 0.8. We also used Monte Carlo simulations to evaluate the adequacy of sampling for the complete microplastic dataset. To do this, we combined the Summer 2009 and Fall 2010 data and fit them to a negative exponential function. We used the rate of change parameter derived from the data (rate = 1.44) to generate 5 sets of randomly deviating exponential distributions with sample sizes between n = 2 and n = 1,000 (4,995 simulations in total). We then calculated and plotted the standard deviation for each distribution.

We computed all statistics using the R statistical environment (version R-2.13.1) [47]. Data were non-normal so nonparametric tests (Mann-Whitney U, Spearman rank correlation) were used. Data from this study are deposited with the California Current Ecosystem LTER DataZoo.

## Results

### Spatial variation in distribution and abundance of plastic debris

The Summer 2009 cruise track covered 4,400 km, with 1,343 km of visual observations, 119 manta tows, and 24 bongo tows. On this cruise, 3,464 pieces of plastic marine debris were sighted through visual observation, 30,518 microplastic particles were collected by manta tow, and 324 microplastic particles were collected by bongo tow. The Fall 2010 cruise track covered 3,800 km, with 28 manta tows that collected 1,572 plastic particles.

**Figure 2:**
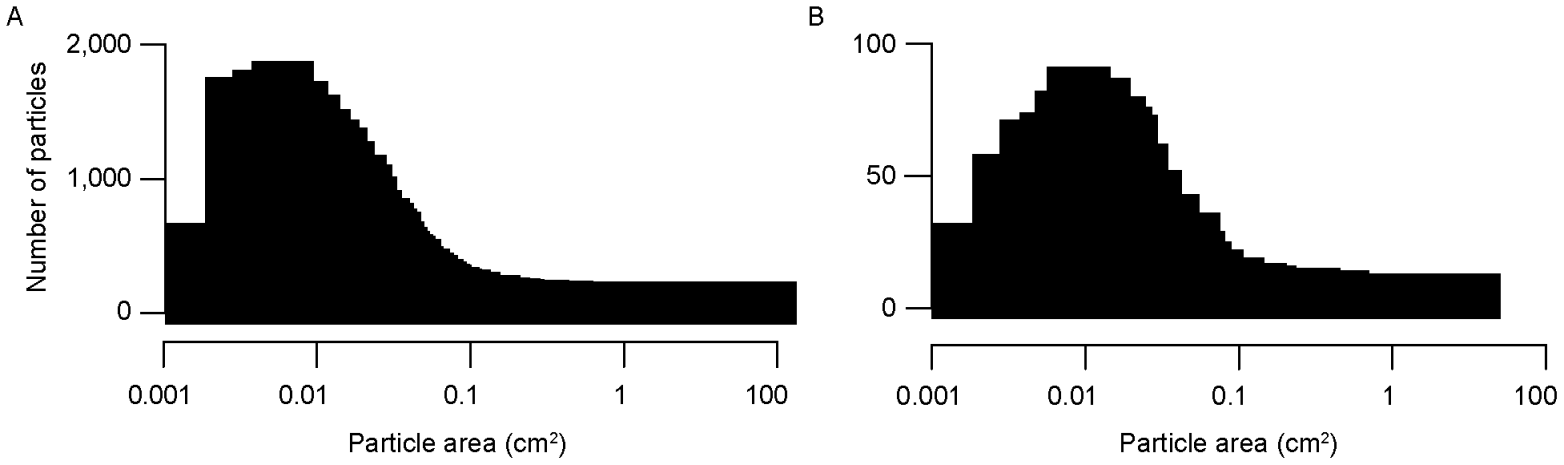
Microplastic size spectra. Histogram of microplastic cross-sectional areas in A) Summer 2009 (n = 30,518) and B) Fall 2010 (n = 1,572). Figure shows all particles collected by manta tow. Visual observations are not included.

**Figure 3:**
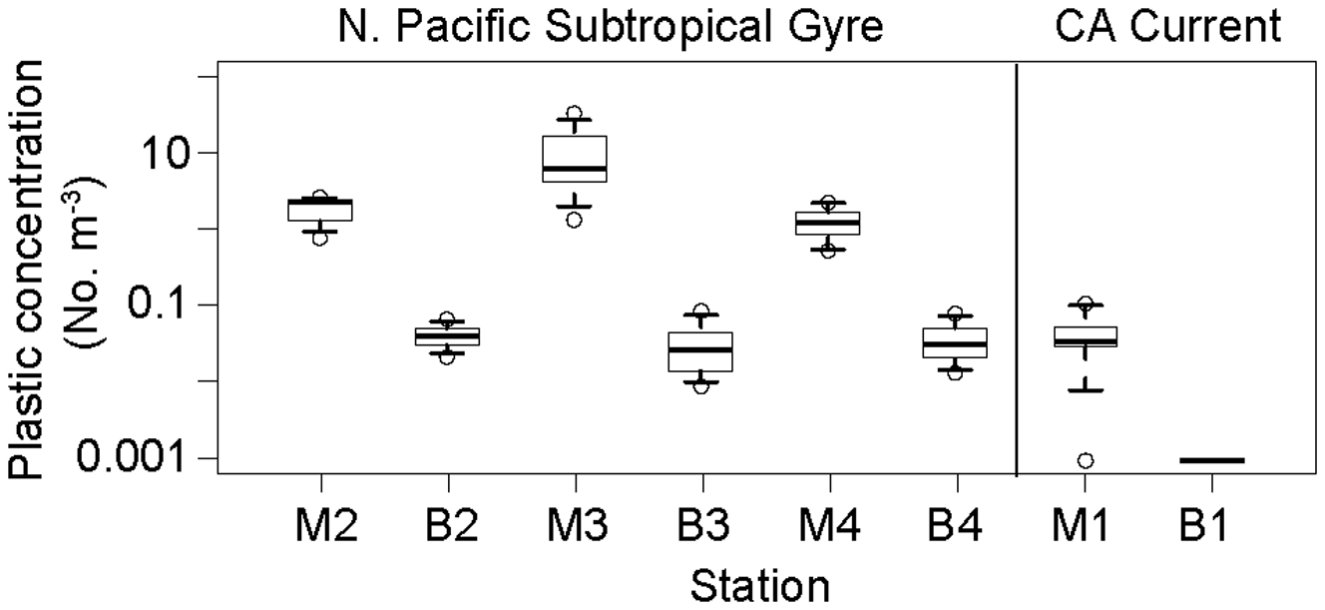
Numerical concentrations of microplastic from neuston samples and sub-surface samples. M indicates manta tows, B indicates bongo tows, and the number refers to the station. Boxes are middle 50% of the data, with the thick line denoting the median. Whiskers indicate 5^th^ and 95^th^ percentile of the data, and hollow circles indicate maximum and minimum values. Sample sizes are n = 8 for each manta tow box plot and n = 6 for each bongo tow boxplot, for a total of n = 32 manta tows and n = 24 bongo tows.

In both years, the highest concentrations of manta-tow collected microplastic were found offshore, rather than adjacent to coastlines (Fig. 1). Median plastic densities, 5^th^–95^th^ percentiles, and maximums are given in Table 1. In both years, the size distribution of particles collected by net tow was skewed towards the smaller end of the size spectrum, with the most abundant particles having an area of approximately 0.01 cm^2^ (Fig. 2). During Summer 2009 at stations in the California Current and NPSG, significantly higher particle densities were found in the neuston than in the integrated water column from the sea surface to 210 m (Fig. 3; Mann-Whitney U test, p<0.01). No subsurface tows were performed in Fall 2010.

**Table 1:** Microplastic numerical concentration (particles m^−2^) in Summer 2009 and Fall 2010.

	Median	95% confidence intervals	Maximum
**Summer 2009**	0.448	0.007–3.211	6.553
**Fall 2010**	0.021	0.002–0.682	0.910

Summer 2009 n = 119, Fall 2010 n = 28.

Plastic concentrations were variable over relatively small spatial scales. For 16 samples taken 10 km apart in a grid pattern, median particle was 0.832 particles m^−2^, but the 5^th^ and 95^th^ percentiles of the data were 0.390–2.023, with the coefficient of variation of 71.2%. In the line sampling pattern with repeated samples taken at stations 18 km apart (Table 2), both visual counts and net sampled concentrations of plastic were variable, with a mean within-station coefficient of variation of 66.3% for the visual samples and 51.4% for the net samples.

**Table 2:** Numerical concentrations of macrodebris and microdebris at four intensively sampled stations in the North Pacific Subtropical Gyre.

Station	Median macrodebris (No. m^−2^)	95% confidence intervals	Median microdebris (No. m^−2^)	95% confidence intervals
1	0.0014	0.0005–0.0036	0.1659	0.0702–0.3486
2	0.0032	0.0008–0.0065	0.3907	0.3245–0.7933
3	0.0015	0.0004–0.0034	2.4321	0.9526–2.8531
4	0.0016	0.0007–0.0026	0.8159	0.3948–1.1450

Data is from line pattern deployed on August 15, 2009 proceeding west from 34°3.4′N, 141°22.4′W (designated by (2) in Fig. 1). The line consisted of 4 stations of 5 repeated manta tows, for a total of 20 manta tows. The four stations were 18 km apart. Visual transect sampling of plastic macrodebris was performed between tow stations, with macrodebris observations for 9 km on either side of given station assigned to that station. Macrodebris concentrations were not statistically different among stations (Kruskal-Wallis test p>0.05). Microdebris concentration from the net tows were statistically different among stations (Kruskal-Wallis test p = 0.002), which was caused by the difference between station 1 and 3 (Nemenyi-Damico-Wolfe-Dunn test, p<0.001). Microplastic concentrations between the other net tow stations were not significantly different (Nemenyi-Damico-Wolfe-Dunn test, p>0.05).

**Figure 4:**
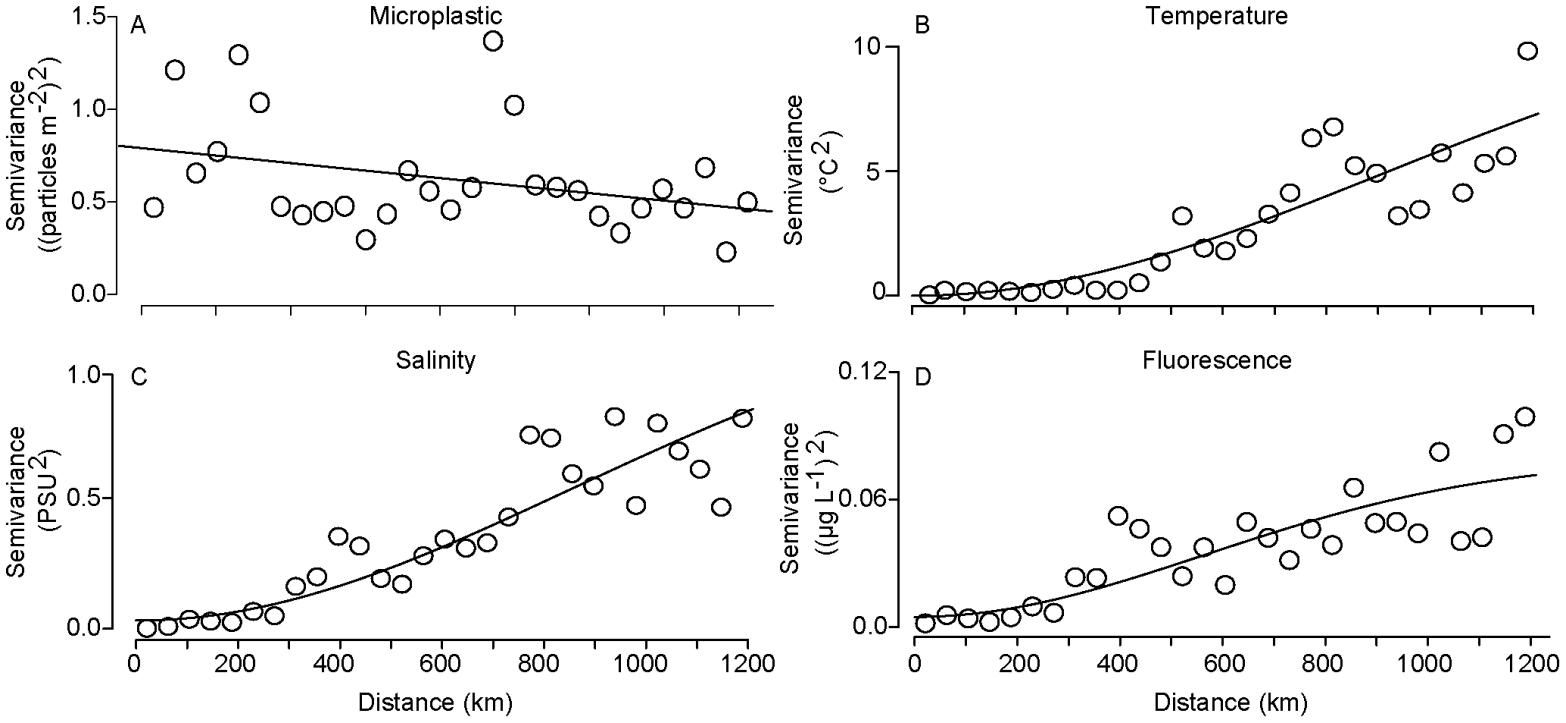
Scale-dependence of variance in microplastic concentration and surface biophysical variables. A) Microplastic numerical concentration; B) Sea surface temperature; C) Sea surface salinity; D) Sea surface fluorescence. Dots are the values of the empirical semivariogram and the lines are a description of the data trends. A is fitted with a linear model, and B–D with Gaussian models. Data are shown for Summer 2009 only since sample size in Fall 2010 was insufficient for this analysis.

Plastic concentrations were also highly variable over the large scale. Over the 2009 cruise track, the semivariance of plastic concentrations was negatively correlated with sample distance (Fig. 4a). A negative correlation suggests that samples that were taken close together were more different from each other than samples that were taken far apart, an illogical result that is most likely an artifact of relatively high variability and low sample size [41]. In contrast, the semivariance of temperature, salinity, and fluorescence all increased with distance between sampling sites, showing the expected result that samples taken close together were more similar to each other than samples taken far apart, though none reached a sill (Fig. 4b–d).

**Figure 5:**
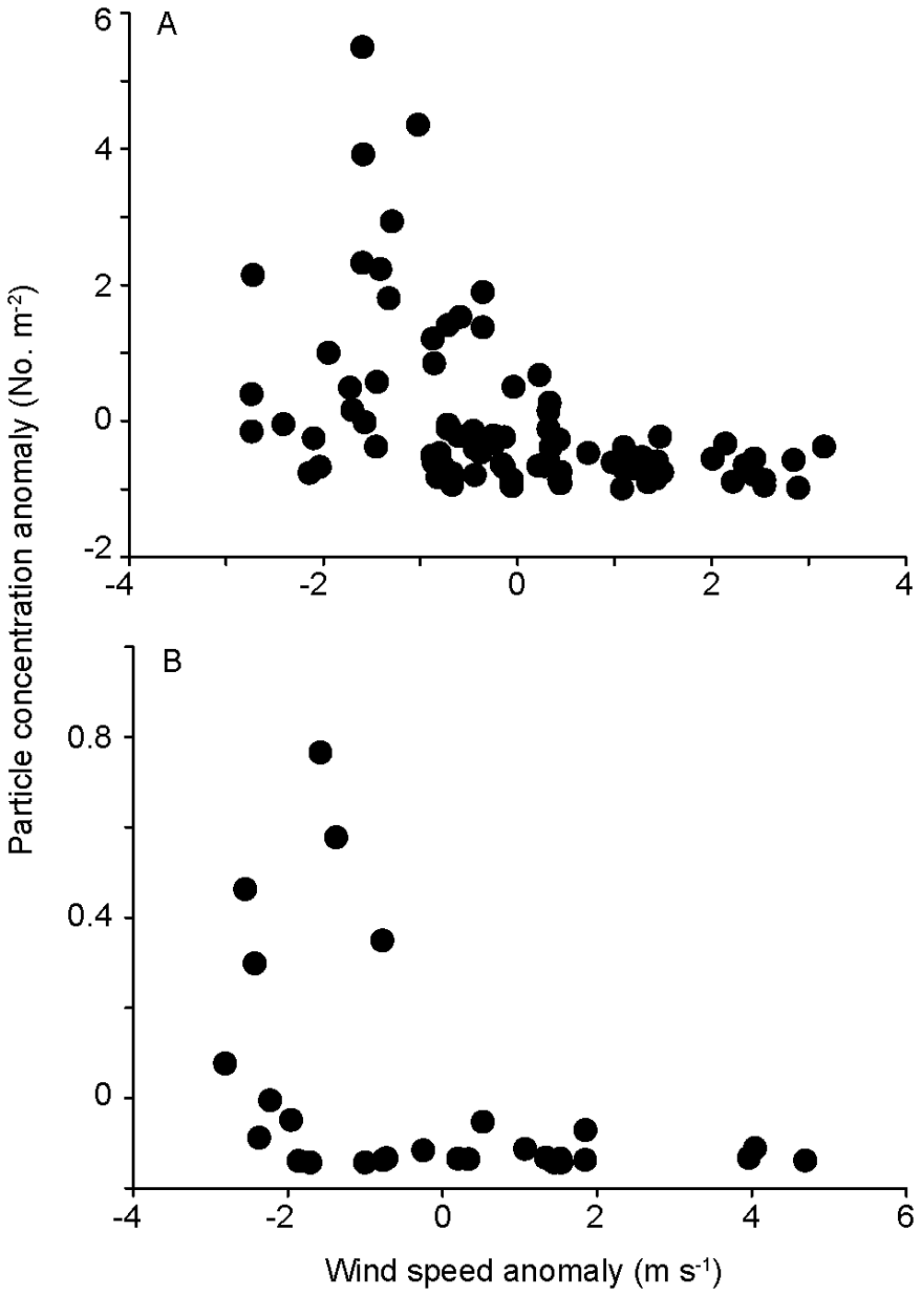
Particle concentration anomalies vs. wind speed anomalies. A) Summer 2009; and B) Fall 2010. Particle density was measured in surface manta net tows, and wind speed recorded by on-ship instrumentation during particle sampling. Sample sizes for Summer 2009 are n = 119 and for Fall 2010 n = 28. No line is shown due to poor fit with both polynomial linear, polynomial quadratic models, with less than 20% of the particle anomaly explained by wind anomalies.

We compared microplastic abundance anomalies (No. m^−2^) and mean hourly wind anomalies (m s^−1^), and found that the highest microplastic abundances were detected only during low-wind conditions (Fig. 5). Spearman's rank correlation coefficient and p-values were rho = −0.567 and p = <0.0001 for Summer 2009, and rho = −0.427 and p = 0.027 for Fall 2010. However, potentially due to high variability, the data were a poor fit to both polynomial linear and polynomial quadratic models, both of which explained less than 20% of the particle anomaly.

**Figure 6:**
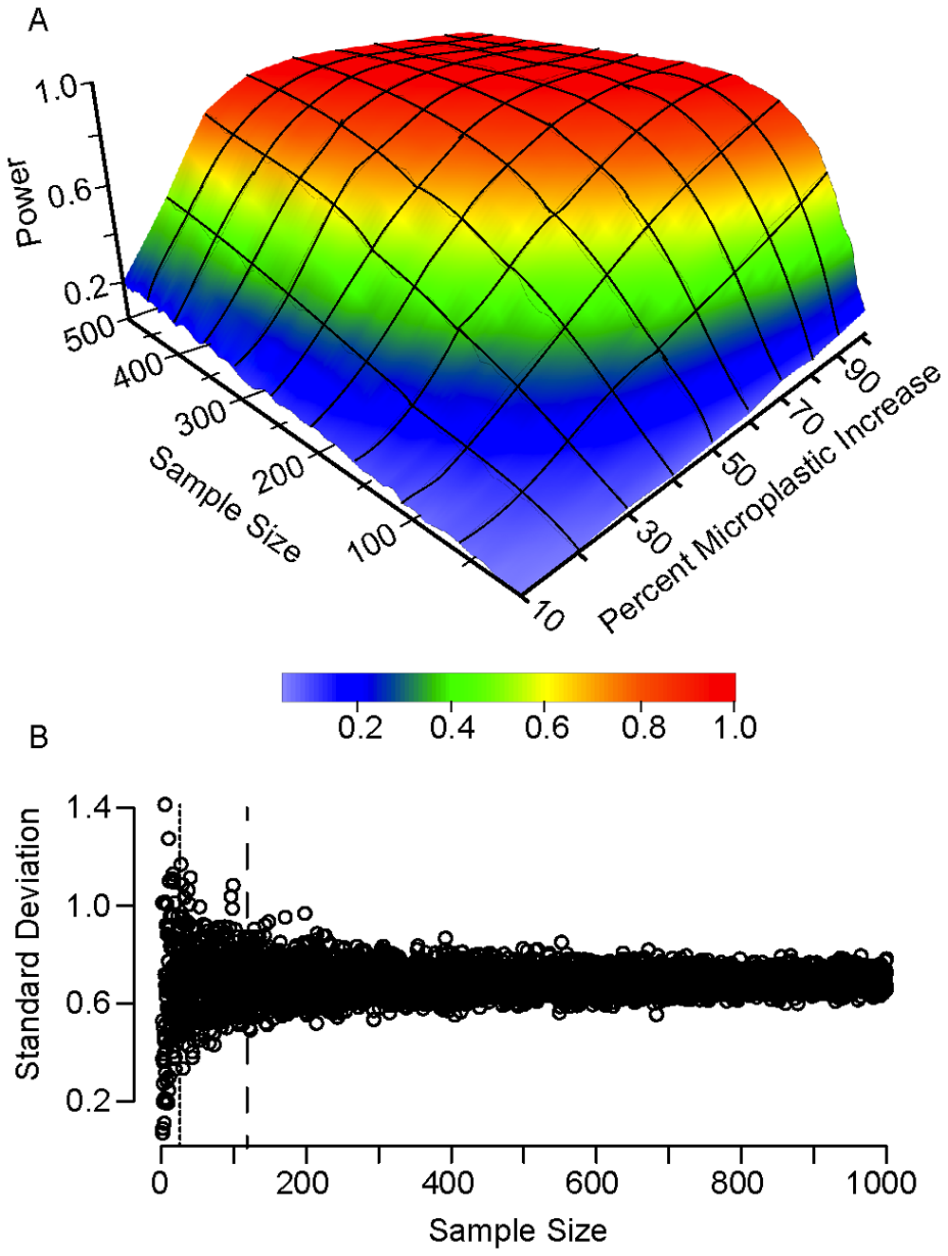
Number of samples necessary to detect changes in microplastic concentration. A) The number of samples necessary (x-axis) to detect a percentage increase in the abundance of microplastic (y-axis) with a certain power (z-axis). For example, using a power of 80%, detection of a 50% increase in microplastic would require a sample size of n = 240. Analysis is based on surface microplastic data from Summer 2009. B) The number of samples necessary to reduce standard deviation of surface microplastic abundance. Dashed line is n = 119, the number of surface microplastic samples collected in Summer 2009. Dotted line is n = 28, the number of surface samples collected in Fall 2010. Analysis is based on surface microplastic data from Summer 2009 and Fall 2010.

High variability may also cause changes in microplastic abundance to be difficult to detect. A power analysis of the 2009 data found that a high number of samples would be needed to detect increases or decreases in microplastic with reasonable probability (Fig. 6a). For example, using a power of 80%, plastic abundance would need to increase by 90% to be detectable with a sample size of n = 100. Under the same adequacy, detection of a 50% increase in microplastic would require a sample size of n = 240. Overall variance may be somewhat reduced by increasing the sample size, but would remain relatively substantial even with increased sampling (Fig. 6b). While a sample size of n = 250 would be preferable, the Summer 2009 sample size of n = 119 was reasonably adequate. However, the Fall 2010 sample size of n = 28 was insufficient to resolve variance.

### Size, shape, and mass of plastic particles

**Figure 7:**
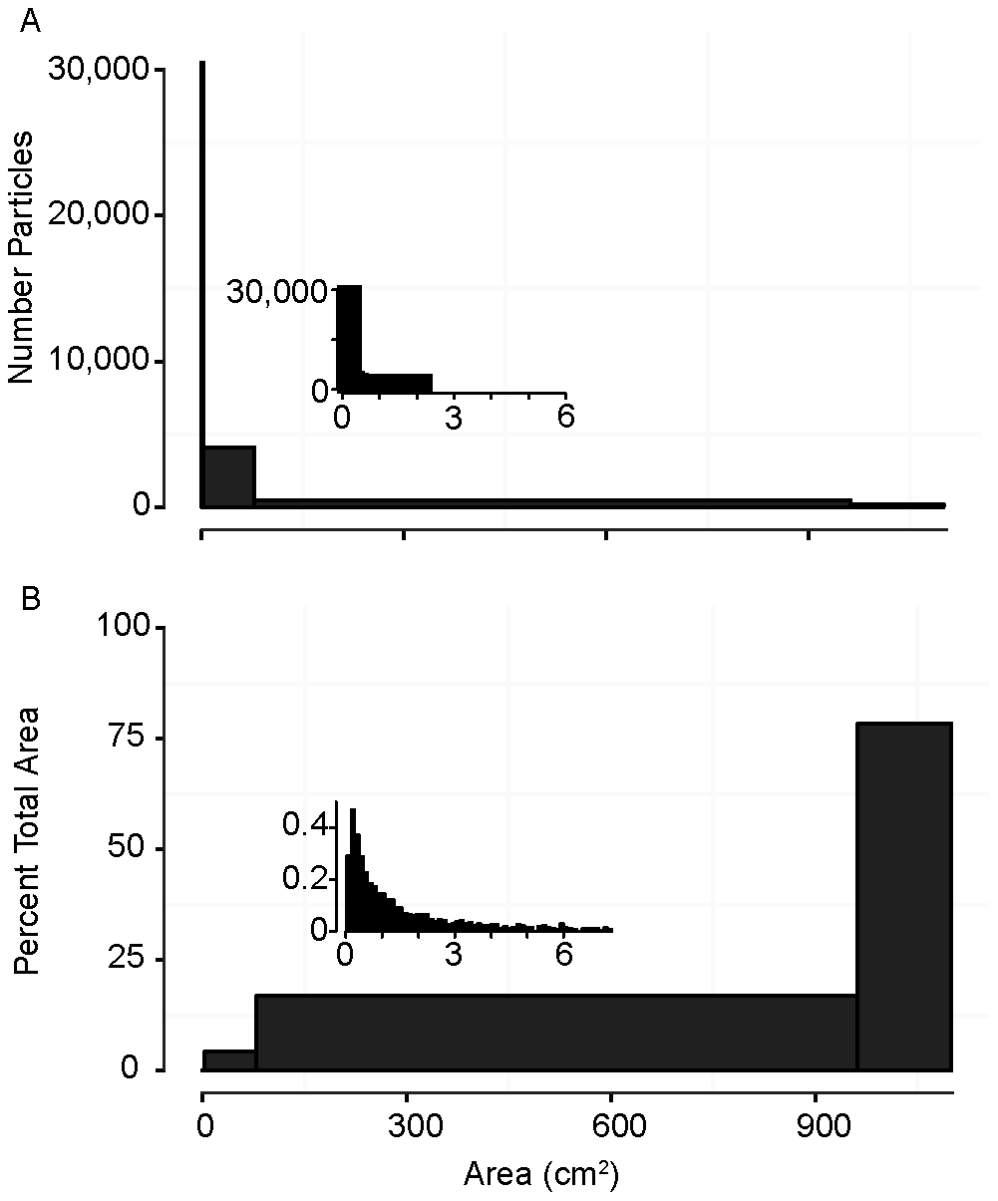
Numerical abundance and percent cross-sectional area of plastic debris by size and water mass. A) Numerical abundance of plastic debris by cross-sectional area; B) Percentage total debris area by cross-sectional area. Insets are an enlargement of the left side of the x-axis from 0 to 6 cm^2^. Includes both net-collected surface microplastic data and visually assessed macroplastic from Summer 2009, for a total of n = 34,233.

In all sections of the cruise track in Summer 2009, plastic debris less than 1 cm^2^ was by far the most numerically abundant (Fig. 7). However, the majority of the two-dimensional area of plastic debris was found in the large, relatively rare items. The sum of the two-dimensional surface area for all plastic debris observed in Summer 2009 was 22.9 m^2^ for the minimum visual estimate, and 14,745.8 m^2^ for the maximum estimated, over the total of 94.4 km^2^ sampled. Therefore, the percentage of ocean physically covered by plastic during this cruise was estimated to range from 2.43×10^−05^% to 0.02%.

**Figure 8:**
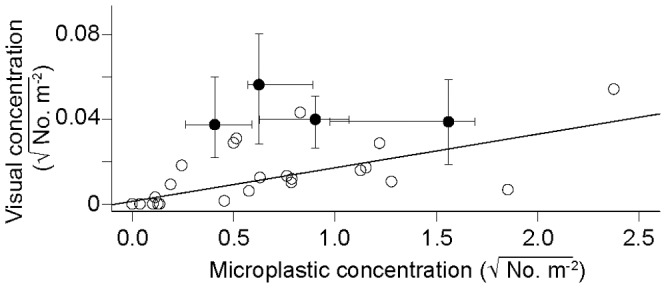
Comparison of plastic debris concentrations from visual and net tow data. Hollow circles indicate stations with visual observations and manta tow stations within 25(n = 23). Solid circles indicate median plastic abundance for visual observations and manta tow stations taken on the line sampling patterns within 9 km of each other. Lines extending from solid circles are bootstrap 95% confidence intervals. Data for solid circles is given in Table 2. Spearman's rank correlation rho = 0.603, p = 0.001. Regression line fit using Theil-Sen single median method.

Plastic concentrations as detected in visual observations and plankton tows within 25 km of each other were positively correlated over the cruise track (Fig. 8, Spearman's rank correlation rho = 0.603, p = 0.001). However, macrodebris and microdebris were not well correlated on smaller (10 km) spatial scales (Fig. 8, filled circles).

**Figure 9:**
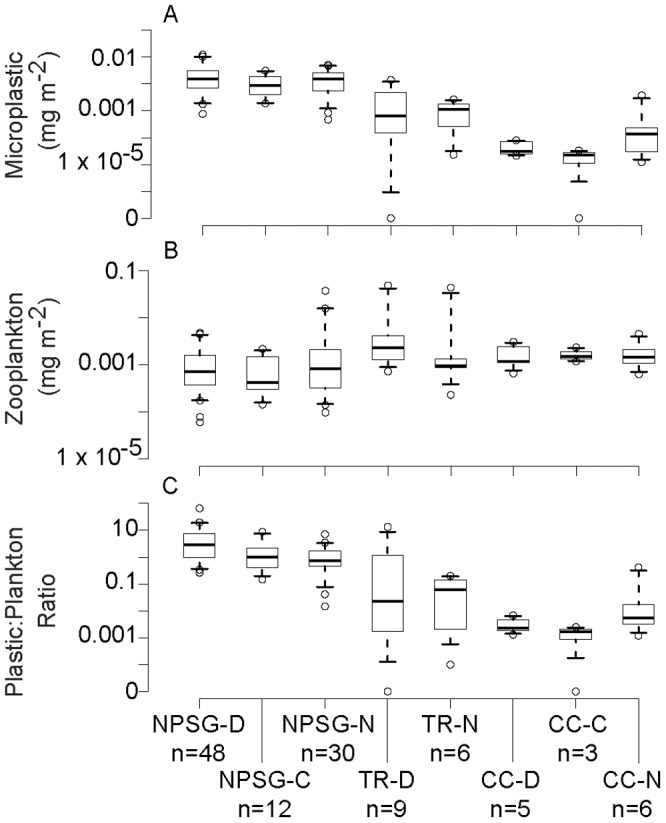
Dry masses of microplastic and zooplankton by location and time of day. A) Dry mass of microplastic; B) Dry biomass zooplankton; C) Ratio of plastic dry mass to zooplankton dry mass. Dry masses are given in mg m^−2^. Boxes are middle 50% of the data, with the line denoting the median. Whiskers indicate 5^th^ and 95^th^ percentiles of the data, and hollow circles indicate maximum and minimum values. Abbreviations are North Pacific Subtropical Gyre (NPSG), transition region (TR), and California Current (CC), but water masses should be considered highly approximate. Time of day is abbreviated to D = day, C = crepuscular, and N = night. Only data from Summer 2009 are shown. Sample sizes are as follows: NPSG-D = 48, NPSG-C = 12, NPSG-N = 30, TR-D = 9, TR-N = 6, CC-D = 5, CC-C = 3, CC-N = 6.

Ratios of dry plastic mass to neustonic zooplankton biomass were positively correlated with plastic mass (Fig. 9, Spearman's rank correlation rho = 0.614, p<0.001) but not zooplankton dry mass (rho = −0.133, p = 0.150). These ratios were not significantly different by time of day with the exception of the NPSG day vs. night samples (pairwise Wilcoxon rank sum test, p<0.001).

## Discussion

This study documents widespread, though spatially and temporally variable plastic pollution in the northeast Pacific Ocean. The median concentration of microplastic (0.448 particles m^−2^) found in Summer 2009 was higher than maximum values from past studies in the NPSG of 0.3168 particles m^−2^ [48] and 0.3343 particles m^−2^ [49]. The maximum concentration reported in this study (6.553 particles m^−2^) is an order of magnitude greater than the maximum of 0.580 particles m^−2^ reported from the North Atlantic Subtropical Gyre [50]. The extremely high concentrations of microplastic found in this study may be due to calm, glassy conditions that allowed less buoyant particles to rise to the air-sea interface [20] and due to a sampling scheme that deliberately targeted high-plastic areas. Nonetheless, our finding of a median plastic concentration nearly double that of the highest plastic concentrations found in past studies suggests that microplastic contamination of the NPSG mixed layer may be more substantial than previously thought.

Our ability to detect high-plastic areas varied with methodology. While visually-detected macroplastic was broadly correlated with net-tow-caught microplastic, these methods did not necessarily correlate on smaller (10 km) scales, and stations with substantial microplastic did not necessary also contain substantial macroplastic. This lack of correlation may be caused by limitations in the visual observation technique, differences in the forces that move different size classes of debris, and high sample variability. Visual estimates may have been hampered by the extremely high densities of marine debris in the smallest observable size class (2–10 cm), leading to an underestimate of overall visually observable debris. In addition, there is debate about how best to categorize the size of debris. In this study, we used only three broad size classes, while others have used a larger number of categories, each covering a smaller range [21]. Since additional scales of classification require more time to record, a tradeoff must be made between the number of size classes recorded and an overall estimation of plastic abundance. In the future, visual underestimates in density would likely be resolved by restricting observations to certain types or size classes of macrodebris, or by observing over smaller defined strip widths along the cruise track [21]. There may also be a spatial mismatch in overall debris distribution due to the higher windage of macrodebris compared to microdebris. For example, in a study of estuarine benthic debris, Browne et al. (2010) found that low density macrodebris moved with the wind, but low density microdebris did not [19]. High sample variability may have been enhanced by a limited number of comparable visual and net tow observations, which a different study design could mitigate.

The calm conditions in Summer 2009 may also have contributed to our finding that microplastic was more abundant in the neuston than in the subsurface water column, since reduced winds resulted in less mixing of plastic particles from the neuston into the subsurface zone. This is supported by our finding that the highest particle concentrations were only detected in low-wind conditions. A similar pattern was found in the North Atlantic Subtropical Gyre by Kukulka et al. (2012), who estimated that 54% of plastic pieces are mixed below surface tow depths under average wind conditions. However, this pattern may not hold outside the subtropical gyres. For example, in the relatively windy California Current, Doyle et al. (2011) found more debris on the surface than in the subsurface water column [51]. We should note that the difference between surface and subsurface microplastic concentrations may be larger than presented here, since the subsurface bongo nets used finer mesh (202 µm) than the surface-sampling manta nets (333 µm). The calm conditions over much of Summer 2009 also allowed the clear visual observation of debris, including small pieces very close to the ship. Ryan (2013) determined the maximum detection rate to be 10–20 m from the ship, partially because the bow wave of the ship, combined with the sea conditions can hinder observations of debris in the 0–10 m distance bin [21]. However, because of the sea conditions experienced during the Summer 2009 cruise, we were able to observe within this closest distance bin, although future studies should consider vessel used and the environmental conditions encountered when determining observation methodology.

Our use of a non-closing net and oblique tow technique means that the depth at which subsurface particles were collected is not known. However, 4.8% of non-vertically migrating planktivorous mesopelagic fishes collected on the same cruise had plastic particles in their stomachs [52]. Since these particles were too large to have been ingested by these fishes' prey, some plastic particles may be sinking outside the euphotic zone. Sinking may be influenced by biofouling-induced changes in density [53], although plastic has not been documented to be a significant component of the material collected in sediment traps [50]. Both these studies and the findings reported here emphasize the need for more detailed observations of the vertical distribution of plastic during various wind conditions.

To our knowledge, this study is the first size spectrum to include both micro- and macrodebris. Our finding of an overall negative exponential relationship between size and particle abundance has been found in some, but not all previous studies of sea surface microplastic [54]. Numerically dominant small particles may be more important for risks that depend on encounter frequency, such as ingestion, microbial growth, and ecotoxicity [7], [55]. However, large items may be more important for risks that are surface area dependent, such as entanglement [56] and the transport of potentially invasive species [57]. Such risks may potentially be mitigated by targeted removal of large objects.

Past studies have used the ratio of dry plastic mass to zooplankton biomass to assess the risk of debris ingestion by marine planktonic filter feeders [49], [58], [59]. This approach, dubbed the “plastic to plankton ratio,” is problematic for a number of reasons described in Doyle et al. (2011), such as the high variance of both plastic and zooplankton in space and time, selective sampling by nets, and selective feeding by zooplankton [51]. Our data confirm that this ratio is conflated with large-scale patterns of plastic abundance and, to a lesser extent, with time of day, though it should be noted that our sample size in certain areas (e.g., the California Current at crepuscular times) was very low. Though the ratio was not significantly correlated with zooplankton biomass over our sampling region, ephemeral high-biomass events may influence it. For example, ratios in the transition region during our sampling period spanned seven orders of magnitude due to a salp aggregation and spatially patchy microplastic, obscuring both the relative abundances of plankton and plastic.

However, the ratio of dry plastic mass to zooplankton biomass may be useful in assessing microplastic remediation schemes. Multiple plans to remove microplastic from the ocean have been proposed and received substantial public attention [60]–[62]. Using the ratio to examine the relative abundance of microplastic and plankton make the ecological ramifications of large-scale filtration of the neuston more apparent. For example, based on the median NPSG ratio of 1.368, approximately 731 mg of dry zooplankton biomass would be removed from the NPSG for each gram of plastic removed. This corresponds to approximately 330 mg of carbon removed, assuming carbon content is 0.40 of total zooplankton dry mass [63]. Since overall productivity in the NPSG is estimated to be only 473 mg C m^−2^ day^−1^ [64], a remediation scheme that removed significant amounts of microplastic would likely have a substantial impact on surrounding plankton standing stocks and, consequently, on nutrient dynamics.

Public concern about plastic debris in marine ecosystems has grown in recent years, resulting in several governmental and non-governmental reports [1], [65]–[67]. More recently, the 2011 Tohoku tsunami [68] and 2012 reauthorization of the United States Marine Debris Research, Prevention, and Reduction Act have raised the profile of this issue even more. However, the efficacy of changes in public policy, industry, or consumer behavior will be difficult to determine without accurate assessments of debris abundance. This will require spatial variability to be taken into account, both so that there is sufficient power to resolve trends and so that the differing spatial patterns between size classes of debris can be resolved. The power analyses presented in this study (Fig. 6) suggests that a large number of samples are needed to detect trends – for example, detecting a 50% increase in microplastic with 80% probability would require 250 neuston samples. Even a snapshot of plastic abundance requires more than 50 neuston samples. Logistical limitations on sampling design (e.g., limited ship time, sampling processing) will therefore make basin-scale debris assessment difficult.

Future surveys might limit this variability through a tighter focus on specific objectives. For example, surveys concerned with invasive species transport might investigate large objects more likely to carry fouling communities, or surveys concerned with biotic interactions might target submesoscale features with high levels of both biological activity and debris, such as fronts or eddies. Sampling limitations may also be mitigated by working with existing oceanographic monitoring programs such as the Hawaiian Oceanographic Time-Series (HOTS), the California Cooperative Oceanographic Fisheries Investigations (CalCOFI), or Sea Education Association (SEA). Alternate methodologies, such as the at-sea enumeration method used by SEA [50], may also be useful. Though the challenges of monitoring are considerable, it is clear that microplastic is now pervasive in the NPSG ecosystem and should be considered when assessing ecosystem health and function.
